# Motor Unit Abnormalities in *Dystonia musculorum* Mice

**DOI:** 10.1371/journal.pone.0021093

**Published:** 2011-06-15

**Authors:** Yves De Repentigny, Andrew Ferrier, Scott D. Ryan, Tadasu Sato, Rashmi Kothary

**Affiliations:** 1 Ottawa Hospital Research Institute, Ottawa, Ontario, Canada; 2 Department of Cellular and Molecular Medicine and the Department of Medicine, University of Ottawa, Ottawa, Ontario, Canada; Brigham and Women's Hospital, Harvard Medical School, United States of America

## Abstract

Dystonia musculorum (*dt*) is a mouse inherited sensory neuropathy caused by mutations in the dystonin gene. While the primary pathology lies in the sensory neurons of *dt* mice, the overt movement disorder suggests motor neurons may also be affected. Here, we report on the contribution of motor neurons to the pathology in *dt^27J^* mice. Phenotypic *dt^27J^* mice display reduced alpha motor neuron cell number and eccentric alpha motor nuclei in the ventral horn of the lumbar L1 spinal cord region. A dramatic reduction in the total number of motor axons in the ventral root of postnatal day 15 *dt^27J^* mice was also evident. Moreover, analysis of the trigeminal nerve of the brainstem showed a 2.4 fold increase in number of degenerating neurons coupled with a decrease in motor neuron number relative to wild type. Aberrant phosphorylation of neurofilaments in the perikaryon region and axonal swellings within the pre-synaptic terminal region of motor neurons were observed. Furthermore, neuromuscular junction staining of *dt^27J^* mouse extensor digitorum longus and tibialis anterior muscle fibers showed immature endplates and a significant decrease in axon branching compared to wild type littermates. Muscle atrophy was also observed in *dt^27J^* muscle. Ultrastructure analysis revealed amyelinated motor axons in the ventral root of the spinal nerve, suggesting a possible defect in Schwann cells. Finally, behavioral analysis identified defective motor function in *dt^27J^* mice. This study reveals neuromuscular defects that likely contribute to the *dt^27J^* pathology and identifies a critical role for dystonin outside of sensory neurons.

## Introduction

The cytoskeleton is a critical intracellular determinant of cellular morphology and is required for fundamental processes during cell differentiation and function [1], [2], [3]. The Plakin protein family comprises high-molecular weight multi-domain molecules, which include desmoplakin, plectin, envoplakin, periplakin, Microtubule Actin Crosslinker Factor 7 (Acf7/MACF) and dystonin/bullous pemphigoid antigen 1 (dystonin/Bpag1). These proteins all share the plakin domain and various other cytoskeletal domains, allowing for simultaneous cross-linking and modulation of cytoskeletal elements [4]. Mutations in genes encoding plakin proteins lead to axonal outgrowth defects, neuronal degeneration, and tissue fragility [5].

**Figure 1:**
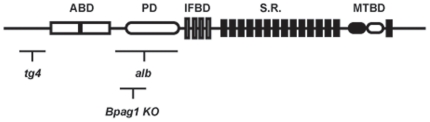
Schematic representation of domains within the dystonin protein and the relative location of mutations for some *dt* alleles. The *dt^tg4^* mutant line has a 45-kb transgene insertion at the 5′ region of the gene, resulting in deletion of coding regions upstream the actin binding domain (ABD). The *dt^Alb^* allele is a spontaneous mutant line resulting in a deletion within the central region of the gene (plakin domain [PD] and intermediate filament binding domain [IFBD]). The *dt^tm1efu^* (Bpag1 knock out [KO]) was generated through homologous recombination. All three *dt* alleles affect all known neuronal and muscle dystonin transcripts and are effectively null mutations. The *Dst* gene includes coding sequences for an ABD, PD, IFBD, Spectrin Repeats (S.R.), and Microtubule Binding Domain (MTBD).

The importance of dystonin/Bpag1 is highlighted in the *dystonia musculorum* (*dt*) mouse. The *dt* mice were originally described as a spontaneously arising mutant with a severe movement disorder [6]. To date, several *dt* mouse models exist including numerous spontaneous mutants (e.g. *dt^24J^, dt^27J^, and dt^Alb^*), 4 chemically induced mutants, 1 targeted allele (*dt^tm1Efu^*), and 1 transgene insertion (*dt^Tg4^*) [7]. In only three *dt* mutations (*dt^Tg4^, dt^tm1Efu^, and dt^Alb^*) have the mutations been characterized [8], [9] (Fig. 1).

The *dt* phenotype, manifesting at ∼2 weeks postnatal, is characterized by loss of limb coordination and abnormal posturing of limbs and trunk. The disease progresses rapidly and *dt* mice die in the third week of life of unknown causes. *Dystonin/Bpag1* encodes alternatively spliced epithelial, neuronal and muscle isoforms [10], [11], [12], [13], [14]. While the epithelial isoform dystonin-e/Bpag1e acts as an autoantigen in the human skin blistering disease bullous pemphigoid, the loss of function of dystonin neuronal and muscle dystonin/Bpag1 isoforms are associated with the *dt* phenotype and underlying pathology in the mouse mutant. The domain architecture of neuronal isoforms can be divided into several parts: an N-terminal actin binding domain, a plakin domain, a central rod region consisting of spectrin-like repeats, a C-terminal region harboring a pair of EF-hand calcium binding motifs and a microtubule binding domain consisting of a GAS2 homology domain as well as a Gly-Ser-Arg repeat region [14], [15] (Fig. 1). Dystonin/Bpag1 is expressed throughout mouse development predominating in neurons of cranial and spinal sensory ganglia [16]. As well, dystonin/Bpag1 is expressed in the extrapyramidal motor system, cerebellum and motor neurons [16].

Among the pathological features of various *dt* mouse alleles, sensory neuron degeneration is most apparent. Although the mechanisms mediating degeneration are unclear, much is known about *dt* pathology. Early studies reported the presence of neurofilament (NF) axonal swellings along nerve fibers and a gradual loss of sensory nerve fibers in both the central and peripheral nervous system [6], [17], [18], [19]. In addition, eccentricity of nuclei and dispersement of the rough endoplasmic reticulum have been reported in neurons from mice expressing various *dt* alleles [6], [18], [20], [21]. Moreover, ultrastructure analysis demonstrated the accumulation of organelles within axonal swellings and disorganization of NF and microtubule networks [19], [22]. Indeed, the disorganization of cytoskeletal networks likely contributes to the bidirectional impairment of axonal transport in *dt* sciatic nerves [23].

In addition to the sensory neuron pathology, some motor neuron defects have also been reported in *dt* mice although the importance to pathology has not been elucidated [24], [25]. Improper placement of nuclei and axonal swellings were detected in the ventral root and in the spinal ventral horn at 13 days postnatal [18], [24]. Moreover, an abnormal accumulation of hyperphosphorylated intermediate filaments is evident in the perikarya and proximal regions of axons of spinal motor neurons in *dt* mice [24]. Examination of *dt* motor neurons revealed accumulation of alpha-internexin, peripherin and NF proteins in axonal swellings and aberrant translocation of alpha-internexin [25]. Finally, motor tests have indicated functional impairment in motor activity, exploration, motor coordination, limb coordination, and postural reflexes [26], [27]. Despite these observations, the previous studies fall short of evaluating the impact of motor neuron abnormalities on neuromuscular/NMJ function/pathology in *dt* mice.

In the present study, we have performed a systematic investigation of motor neuron pathology in the spontaneous mouse mutant *dt^27J^*. We provide evidence for neuromuscular defects in the *dt^27J^* mouse mutant that likely contribute to the overall neurological phenotype.

## Materials and Methods

### Mice

*dt^27J^* mice have a mutation within the *dystonia musculorum* locus which occurred in the congenic strain B10.PL(73N)/Sn. These mice were originally identified at The Jackson Laboratory (Bar Harbor, ME) and breeding pairs were obtained from them. The *dt^27J^* allele was subsequently maintained in our vivarium facility by mating heterozygous mice. At 1–2 weeks of age, homozygous *dt^27J^* mice have difficulty in walking, mainly due to their limb incoordination phenotype. By the third week of life, *dt^27J^* mice succumb to the disease and die. Mice were genotyped by polymerase chain reaction amplification of genomic tail DNA using the oligonucleotides (RAS520) sense 5′ GGA TCT GCC CGA CTT TCT GGG 3′ and (RAS521) antisense 5′ CCA AGG TTC ATT GCC TCC GTC 3′. These primers amplify a 400 nucleotide fragment on the wild type allele and a 450 nucleotide polymorphic fragment on the *dt^27J^* allele.

### Ethics Statement

All experimental protocols on mice were approved by the Animal Care Committee of the University of Ottawa. Care and use of experimental mice followed the guidelines of the Canadian Council on Animal Care.

### Spinal motor neuron quantification

Wild type (n = 5) and *dt^27J^* (n = 5) pups at postnatal day (P)15 were anesthetized with tribromoethanol (Avertin) and perfused transcardially with 3 ml of phosphate buffered saline (PBS) followed by 10 ml of 4% paraformaldehyde (PFA) in PBS. The lumbar L1 spinal cord region was collected and embedded into paraffin, cut in totality in serial sections of 6 µm thickness, deparaffinized in toluene and rehydrated in an alcohol gradient followed by deionized water. Sections were stained with Pyronine Y, washed with water, dehydrated in an alcohol gradient, and finally cleared with toluene and mounted with a coverslip using Permount (Fisher). Sections were examined by light microscopy using a Zeiss Axioplan microscope equipped with a digital camera. Alpha motor neurons were identified based on their location in the gray matter that forms the ventral horn and their large size (nuclear diameter >9–10 µm; cell body diameter >20 µm). Gamma motor neurons (nuclear diameter 7–9 µm) and interneurons (nuclear diameter <6 µm) were identified by their respective nuclear diameters [28], [29], [30]. Alpha motor neurons, gamma motor neurons, and interneurons were counted in every 10^th^ section in the ventral horn of the L1 spinal cord. For the quantification of eccentric alpha-motor nuclei, only L1 ventral horn alpha-motor neurons with distinct nuclei (diameter >9–10 µm) and nucleoli were included.

### Antibodies

Eleven primary antibodies were used for immunohistochemistry (IHC) in this study (for details, see Table 1). Secondary antibodies used have been summarized in Table 2.

**Table 1:** Primary Antibodies.

Antiserum	Immunogen	Source(cat. no.)	Working dilution	Specificity
Rabbit polyclonalanti-internexin,alpha	Full-length recombinantrat alpha-internexinfused to E. Coli Trp E.	Chemicon International,Temecula, CA (AB5354)	1∶250 (I)1∶1000 (W)	Alpha internexin(66 KDa)
Rabbit polyclonalanti-laminin	Protein purified fromthe basement membraneof Englebreth Holm-swarm (EHS) sarcoma (mouse)	Abcam (ab11575)	1∶800 (I)	Laminin is composed of one A chain (400 KDa), one B1 chain (215 KDa),
Anti-myosineHeavy chainType 1	MyHC (Human)partially purified, pyrophosphate extracted (22 year quadriceps)	Developmental StudiesHybridoma Bank(A4.840)	ud (I)	Adult myosin (200 KDa)
Anti-myosineHeavy chainType 2A	Purified myosinorigin from bovinesubcutaneous muscle	Developmental StudiesHybridoma Bank(SC-71)	ud (I)	Immunoblotting (2A myosin heavy chain)
Anti-myosineHeavy chainType 2B	Purified myosinbovine skeletal muscle(fetal, 3 months)	Developmental StudiesHybridoma Bank(BF-F3)	ud (I)	Recognized 2B myosinheavy chain
Anti-myosineHeavy chainType 2X	Myosin co-absorbedwith an adjuvant peptide to gold particles rabbit retractor bulbi muscle	Developmental StudiesHybridoma Bank(6H1)	ud (I)	Immunoblotting (2Xmyosin heavy chain) from rat and rabbit limb muscles (200 KDa)
Mousemonoclonal anti-neuron-specific nuclear protein (NeuN)	Purified cell nuclei frommouse brain	Chemicon International,Temecula, CA(MAB377B)	1∶100 (I)1∶200 (W)	It recognizes 2-3 bandsin the 46–48 KDa range(manufacturer's technical information).
Mousemonoclonal anti-neurofilament RT-97 specific for phosphorylated NF-H	Wistar ratneurofilaments	Developmental StudiesHybridoma Bank (RT-97)	1∶3 (I)	It recognizes a band at 200 KDa corresponding to NF-H protein (manufacturer's technical information).
MouseMonoclonal anti-synaptic vesicle 2 (SV2)	Synaptic vesicleProtein 2	Developmental StudiesHybridoma Bank (SV2)	1∶100 (I)	It recognizes a band at∼95 KDa (manufacturer's technical information)
MouseMonoclonal anti-dystrophin (C-terminus, clone Dy8/6C5)	Synthetic polypeptide re-presenting the last 17 amino acids at the car-Boxy terminus of the human dystrophin molecule (SSRGRNTPGKPMREDTM)	Vector Laboratories(VP-D505)	1∶20 (I)1∶10–1∶25 (W)	Skeletal muscle, singleStrong band at ∼400 KDa (manuf0acturer's technical information).
Mousemonoclonal anti-pan-axonal neurofilaments	Pan-axonalneurofilament	Covance(SMI-312R)	1∶1000 (I)1∶1000 (w)	SMI-312 is a specific marker for axons in tissue sections and cultures.

(I), Immunohistochemistry, (W), Western blot, ud (undiluted).

**Table 2:** Secondary Antibodies.

Secondaryantibodies	Company	Dilution
Goat anti-mouse 555	Invitrogen	1∶500–1∶2000
Goat anti-rabbit 555	Invitrogen	1∶200
Donkey anti- rabbit 488	Invitrogen	1∶500
Goat anti-mouse 488	Invitrogen	1∶200

### Immunofluorescence

L1 spinal cord segments were collected from wild type (n = 3) and *dt^27J^* (n = 3) pups at P15 for cryostat sections of 6 µm thickness. Sections were air dried at room temperature (RT) then rinsed in PBS and fixed in 4% PFA. P15 wild type and *dt^27J^* mice were perfused and fixed in 4% PFA and brain sagitally sectioned at a thickness of 10 µm. Sections were rinsed twice in PBS, followed by incubation in blocking solution (10% goat serum and 0.3% Triton-X100 in PBS) for 45 min at RT. The sections were incubated overnight at 4°C with primary antibody in blocking solution with the exception of anti-alpha internexin which, was administered for 2 h at RT. After incubation, slides were rinsed in PBS three times and then incubated with appropriate secondary antibodies in blocking solution for 1 h at RT. Sections were then rinsed in PBS and incubated for 15 min with 3,3′-diaminobenzidine (DAPI) (1∶10,000) in blocking solution. Finally, slides were rinsed in PBS and slides were mounted with Fluorescent Mounting Medium (Dako) and examined under a fluorescent microscope.

### Sample preparation for electron microscopy

Wild type (n = 3) and *dt^27J^* (n = 3) mice were anesthetized at P15 via intraperitoneal injection of tribromoethanol. Mice were perfused transcardially with 3 ml of PBS followed by 10 ml of Karnovsky's fixative (4% PFA, 2% glutaraldehyde, and 0.1 M cacodylate buffer in PBS, pH 7.4). Ventral and dorsal roots of spinal nerves were collected under a stereomicroscope. Samples were fixed for 4 h in Karnovsky's fixative at 4°C. Samples were subsequently washed 2 times in 0.1 M cacodylate buffer for 1 h and once overnight at RT. After 1 h of post-fixation in 1% osmium tetroxide in 0.1 M cacodylate buffer at 4°C, all samples were washed twice for 5 min each in distilled water. Samples were dehydrated, infiltrated in spur monomer (Electron Microscopy Sciences) with 3 changes over 24 h at RT and embedded in liquid spur resin at 70°C overnight. Ultrathin sections (70 nm) from ventral and dorsal roots of the spinal nerve were cut in cross-section using an ultramicrotome. Sections were placed on a 300 mesh copper specimen grid and counterstained in 5% uranyl acetate and Reynold's lead citrate, and then observed by transmission electron microscopy. Ventral and dorsal root sections of 0.3 µm were mounted on glass slides and stained with toluidine blue, and then examined by light microscopy using a Zeiss Axioplan microscope equipped with a digital camera. Motor axons were compared between wild type and *dt^27J^* pups, and quantified.

### Quantification of the total number of NMJ endplates in skeletal muscle

Wild type (n = 3) and *dt^27J^* (n = 3) mice were analyzed at P15. For each mouse, one leg was dissected to collect the TA and the EDL muscles for cryostat sections (14 µm) and the other leg was used to isolate individual myofibers. Muscle cross-sections were incubated with primary antibody to dystrophin 10% goat serum and 0.3% Triton-X100 in PBS for 1 h at 25°C. Sections were then washed three times in the same buffer, incubated with the secondary FITC conjugated antibody for 1 h at 25°C, and then washed three times in buffer. Sections were then incubated with alpha-bungarotoxin (Alexa Fluor 488 conjugate, 1∶500, Invitrogen) in PBS at room temperature for 30 min, rinsed twice in PBS, and mounted with Fluorescent Mounting Medium (Dako). NMJs were examined under a fluorescent microscope. The total number of myofibers by cross-section area in the middle of each muscle was multiplied by the number of NMJs by individual myofiber observed. By this approach we have quantified the total number of NMJs for each muscle analyzed.

### Single mouse myofiber isolation

Entire EDL and TA muscles including tendons were collected from wild type (n = 6) and *dt^27J^* (n = 6) mice at P15. Each muscle was digested for a period of 1 h or more in 0.2% collagenase 1 in Dulbecco's minimum essential medium (DMEM) (supplemented with antibiotics [pen/strep]) at 35°C with agitation to ensure that myofibers were loosened from the muscle bulk. After digestion, each muscle was transferred in a Petri dish coated with horse serum containing DMEM medium with 10% horse serum. Single mouse myofiber isolation was performed by trituration using sterile Pasteur pipettes. A set of pipettes with bore diameter of 1 to 2.5 mm was made with a diamond knife and the sharp ends were fire polished. All pipettes were coated with 10% horse serum in DMEM medium [31], [32], [33]. All single myofibers from each muscle were fixed in 4% PFA for 10 min followed by a single wash in PBS and a single wash in 0.1 M glycine/PBS. Single myofibers were incubated with alpha-bungarotoxin in PBS (1∶2500) and DAPI (1∶10,000) for 1 to 4 h with agitation, then washed 3 times for 10 min each. Slides with single myofibers were mounted with fluorescent mounting medium. Fifty myofibers from each muscle were examined under a fluorescent microscope and NMJ endplate quantification was performed. The NMJ endplate morphology was examined in 25 individual wild type myofibers/muscle and also in 25 individual *dt^27J^* myofibers/muscle.

### Immunohistochemistry and evaluation of NMJ endplates from groups of myofibers

EDL and TA muscles were isolated from P15 wild type (n = 5) and *dt^27J^* (n = 5) pups and fixed for 30 min in 2% PFA. After fixation all muscles were washed in 0.75% glycine in PBS at RT and then in PBS. Groups of myofibers were isolated from EDL and TA muscles under a stereomicroscope, then blocked with 10% goat serum in 0.4% Triton X-100/PBS for 30 min at RT, and incubated overnight at 4°C with primary antibodies. Incubation with the secondary antibodies was performed the following day at RT for 1 h. Groups of myofibers were also incubated with alpha-bungarotoxin Alexa Fluor 555 conjugate (1∶10, Invitrogen) for 20 min at RT. Finally, three to four stained myofiber bundles were mounted in Fluorescent Mounting Medium (Dako). Images were taken on a Zeiss LSM 510 confocal microscope with a 40x oil objective, equipped with filters suitable for FITC/Cy3/fluorescence. For the blind quantification analysis, a minimum of 50 NMJs was analyzed for the control and experimental groups. To objectively differentiate between normal and abnormal pre-synapses (axonal swellings) any bulb-like structure exceeding 2 µm in diameter was deemed abnormal. Samples were imaged under identical sample and exposure settings. The number of perforations/endplate defined maturity of the post-synaptic endplate in mice, as previously discussed [34].

### Fiber typing

TA muscles were isolated from P15 wild type (n = 3) and *dt^27J^* (n = 3) pups. TA muscle cryosections (12 µm) were fixed with 4% PFA and washed with PBS, then with buffer (0.3% Triton-X100 in PBS). Muscle sections were blocked with 5% horse serum in PBS for 10 min and then incubated overnight with primary antibodies. Sections were then washed 3×10 min in PBS and incubated for 1 hour with secondary antibodies. Sections were rinsed in PBS, and slides mounted with Fluorescent Mounting Medium (DAKO). Sections were examined under a fluorescent microscope to detect any fiber type grouping.

### Muscle fiber size quantification

Cross-sections of P15 TA muscles (wild type and *dt^27J^*) were stained with hematoxylin and eosin and observed by light microscopy. TA muscle fiber area was measured in µm^2^ (MIRAX program) for 200 myofibers in the central region of the TA muscle for wild type (n = 6) and *dt^27J^* mice (n = 6).

### FluoroJade staining

FluoroJade B (Millipore, MA) staining was performed according to the method of [35]. Briefly, 10 µm sagittal brain sections were dried at 50°C, hydrated with descending grades of ethanol, and finally rinsed with deionized water. The sections were dipped in potassium permanganate solution (0.06% weight/vol) for 10 min, washed with deionized water, and further incubated with FluoroJade B (0.0004% vol/vol) and 0.1% vol/vol glacial acetic acid coupled with DAPI counterstained for 20 min in the absence of light. The sections were washed with deionized water, dried at 50°C, dipped in xylene, and coverslipped with Permount. Sections were then visualized under epifluorescence and photomicrographs captured using a Zeiss Axiovert 200M microscope equipped with AxiovisionLE V 4.8.0.0. FluoroJade positive neurons from two replicates of two independent experiments were quantified by counts of DAPI counterstained nuclei with correction for profile and section thickness according to the method of [36].

### Motor neuron quantification in the brainstem

Wild type and *dt^27J^* brainstem sections (trigeminal nerve of the pons) were immunostained with anti-NeuN primary and anti-mouse 555 secondary antibodies. Sections were then visualized under epifluorescence and photomicrographs captured using a Zeiss microscope equipped with Axiovision LE V4.8.00. Alpha motor neurons were defined as larger NeuN-positive neurons. Motor neurons from two replicates of two independent experiments were quantified by counts of DAPI counterstained nuclei with correction for profile and section thickness according to the method of [36].

### Motor behavior tests at P15

For each motor test, 5 different *dt^27J^* pups and 5 different wild type littermates were evaluated. Five attempts per mouse per test were performed. *Rod test*: Motor performance was evaluated in the mice by measuring their capacity to grasp a flexible rod by their hind legs. Mice were placed at the center of the horizontal rod by their hind legs and the latency to fall was measured. *Righting reflex: dt^27J^* and wild type mice were evaluated for the time to right themselves from a supine position. *Negative geotaxis: dt^27J^ and* wild type mice were placed for 30 sec on a wire grid inclined at 90° to assess negative geotaxis. For methodology, see [37].

## Results

### Motor neurons are not spared in *dt^27J^* mice

In the present study, we focused on the spontaneous mutant *dt^27J^*. While the *dt^27J^* allele does not result in any large genomic rearrangements or deletions at the *dt* locus, it does have a significant impact on neuronal and muscle dystonin transcript expression [7]. It is believed that small rearrangements or deletions, or point mutations causes either reduced transcription or altered transcript stability. Like all known *dt* mice, *dt^27J^* mice are homozygous recessive, heterozygous (*dt^27J/+^*) mice fail to manifest a phenotype. Moreover, the *dt^27J^* and *dt^Tg4^* strains are allelic and failed to genetically complement, indicating that the *dt^27J^* mutation is specific to the dystonin gene [38].

**Figure 2:**
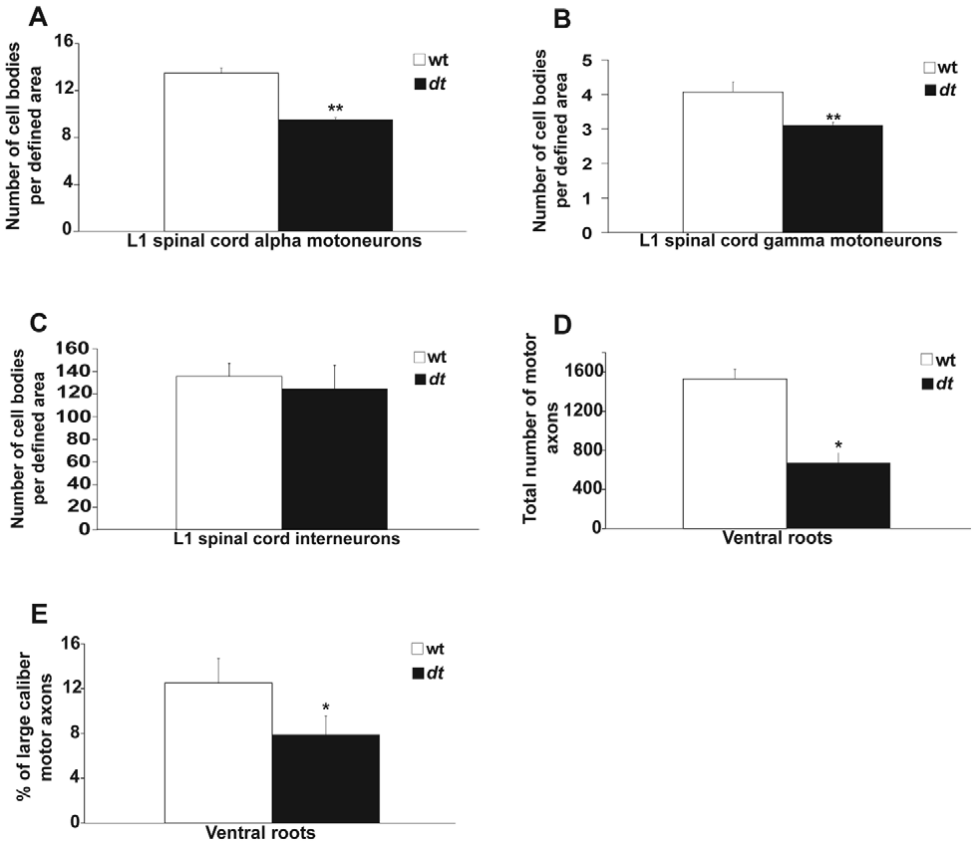
The number of alpha and gamma motor neurons is reduced in dt27J L1 spinal cord. A–B. A graphical representation showing the number of neurons per defined area of the ventral horn of the L1 spinal cord from wild type (n = 5) and severely affected dt27J (n = 5) mice at P15. There is a significant difference in the number of alpha motor neurons and gamma motor neurons between wild type and dt27J mice (**p<0.01, student t-test). C. No significant difference was observed between wild type and dt27J mice in the number of interneurons per defined area of the ventral horn of the L1 spinal cord (p>0.05, student t-test). D. The total number of motor axons is significantly reduced in dt27J mice (*p<0.05, student t-test). E. The percentage of large caliber motor axons is reduced in dt27J ventral roots by comparison to wild type ventral roots (*p<0.05, student t-test).

**Figure 3:**
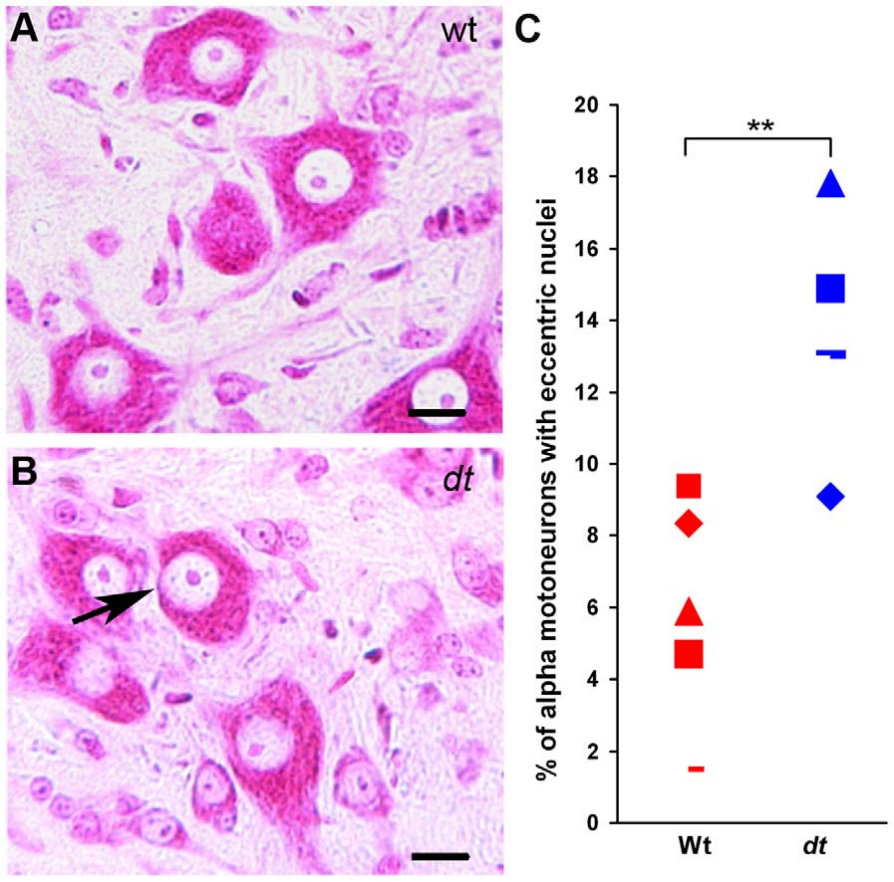
Increase in the number of eccentric alpha motor neuron nuclei in *dt^27J^* L1 spinal cord compared to wild type L1 spinal cord. A. Normal alpha motor neurons were observed by light microscopy after Pyronine Y staining of the ventral horn of the L1 spinal cord from a wild type mouse. B. Eccentric motor nucleus (black arrow) observed in L1 spinal cord from a *dt^27J^* mouse. Scale bar, 10 µm (A, B). C. Dot plot graph showing the percentage of eccentric alpha motor neuron nuclei in the L1 spinal cord region as observed in individual wild type (n = 5) and *dt^27J^* (n = 5) mice (**p<0.01, student t-test).

Previous work has shown that sensory neuron loss is a primary defect in *dt* mice. Here, we set out to determine whether motor neurons are also affected in the homozygous *dt^27J^* mutant mice. Since *dt^27J^* mice are smaller than their wild type counterparts, we decided to measure the number of alpha and gamma motor neurons, and interneurons within a defined area. At P15, a moderate loss of alpha motor neurons and gamma motor neurons (29.40% and 23.58%, respectively) was evident in ventral horns of the L1 spinal cord from *dt^27J^* mice when compared to age matched control wild type mice (Fig. 2A-B). In contrast, there was no significant difference in the number of interneurons at the L1 level between wild type or *dt^27J^* mice (Fig. 2C). Eccentric alpha-motor nuclei in the ventral horn were 2.3 times more abundant at the L1 spinal cord level of *dt^27J^* mice compared to wild type mice at P15 (Fig. 3). Thus, defects are readily observed in alpha and gamma motor neurons from *dt^27J^* mice.

### Neurofilament accumulation in *dt^27J^* motor neuron cell bodies

**Figure 4:**
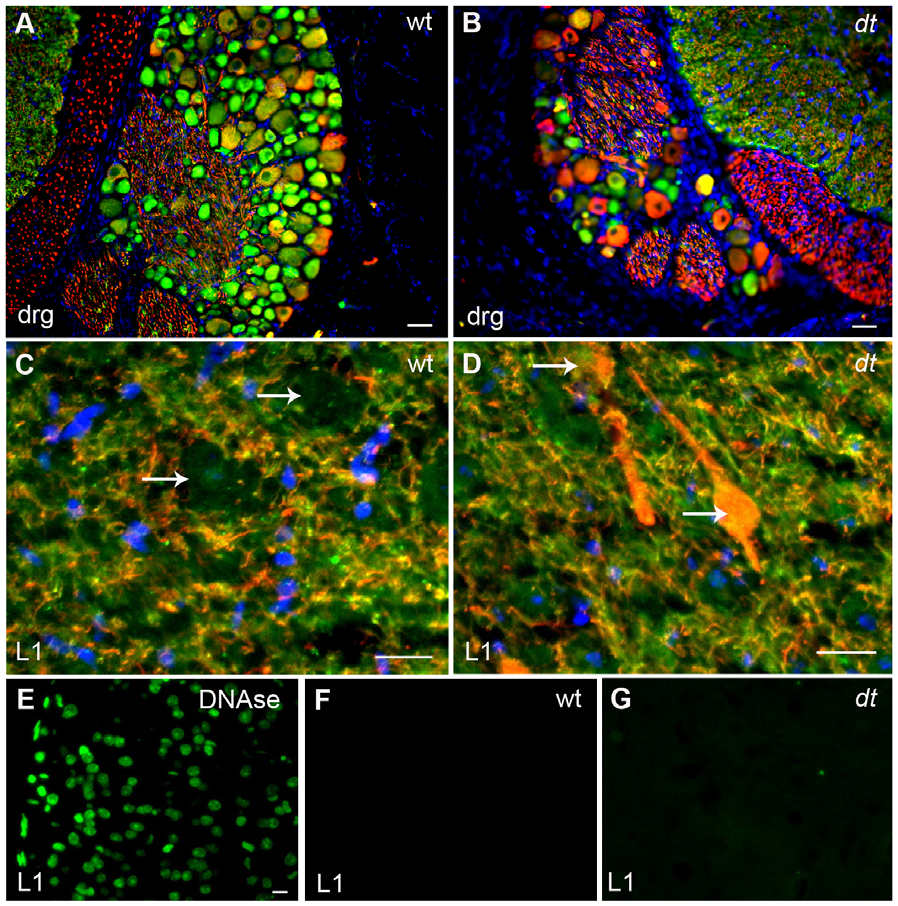
Analysis of neurofilament and alpha-internexin immunostaining in the dorsal root ganglion and ventral horn of the L1 spinal cord of wild type and *dt^27J^* mice. **A**. Alpha-internexin staining (green) in cell bodies within the DRG of wild type mice. RT-97 staining of phosphorylated neurofilaments (red) is limited to a few cells. **B**. The DRG from *dt^27J^* mice is smaller and has fewer ganglion cells per DRG compared to wild type DRGs. RT-97 staining of phosphorylated neurofilaments (red) can be readily seen within the perikarya of many ganglion cells while alpha-internexin staining (green) is also present in a few cells. Scale bar, 10 µm (A, B). **C**. Ventral horn region of the L1 spinal cord from a wild type mouse showing no accumulation of phosphorylated neurofilaments within the perikarya of motor neurons (white arrows). Alpha-internexin staining (green) is observed throughout the dendrites and axons of the motor neurons. **D**. Ventral horn region of the L1 spinal cord from a *dt^27J^* mouse showing abnormal accumulation of phosphorylated neurofilaments within the perikarya (white arrows) of motor neurons. In comparison, alpha-internexin staining (green) is observed in dendrites and axons. Scale bar, 20 µm (C, D). **E**–**G**. TUNEL assay showed no labeling in cells in the L1 spinal cords of WT and *dt^27J^* mice (F–G). DNase treated L1 spinal cords were used as a positive control (E). Scale bar 10 µm.

NF accumulation within sensory neurons of *dt* mice is a pathological hallmark of the disease [18], [24], [25], [39]. To determine if *dt^27J^* sensory as well as motor neurons manifest NF accumulation, we performed immunostaining using anti-alpha-internexin and anti-NF antibodies. Although previous studies have shown that alpha-internexin levels are diminished in DRGs of *dt* mice (Tseng et al., 2006), other work has shown that there is no change in levels [40] Alpha-internexin staining was observed in P15 wild type DRG perikarya, while only a few cells displayed phosphorylated NF (RT-97) staining (Fig. 4A). Although *dt^27J^* DRGs revealed far fewer cell bodies, there were still cells staining for alpha-internexin and a relative increase in the number of cells staining with RT-97 (Fig. 4B). Perikarya of wild type ventral horns were devoid of phosphorylated neurofilament staining (Fig. 4C, white arrows), while alpha-internexin staining was observed in dendrites and axons of ventral horn cells (Fig. 4C). In contrast, *dt^27J^* ventral horn staining revealed an abnormal accumulation of phosphorylated NFs within the perikarya of motor neurons (Fig. 4D, white arrows). This staining was also observed in dendrites and axons of neuronal cells (Fig. 4D). Similarly, alpha-internexin staining was observed in ventral horn cells (dendrites and axons; Fig. 4D). To determine whether the pathology described above translated into motor neuron cell death, we conducted TUNEL staining using L1 spinal cord tissue sections of wild type and *dt^27J^* mice. This assay did not reveal any TUNEL positive cells in the L1 spinal cords of *dt^27J^* mice (Fig. 4E-G). Thus, in addition to sensory neurons, motor neurons from *dt* mice also display abnormal accumulation of NFs.

### *dt^27J^* dorsal and ventral spinal root axons are amyelinated and reduced in number

**Figure 5:**
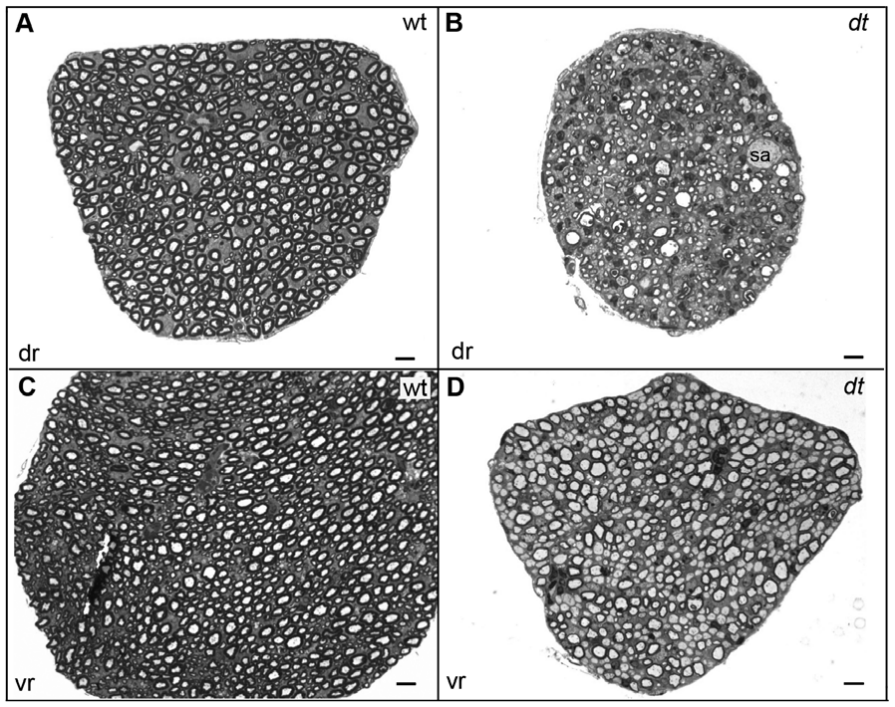
Defects in *dt^27J^* ventral motor and dorsal sensory spinal roots. Toluidine blue staining of transverse sections of dorsal (dr) (**A**–**B**) and ventral (vr) roots (**C**–**D**). **A.** Dorsal sensory root from wild type mice showing many myelinated axons. **B.** Dorsal sensory root from *dt^27J^* mice showing several abnormalities including fewer axons, axons undergoing degeneration, and axonal swellings (sa). The *dt* dorsal sensory root is also smaller than the wild type counterpart. **C.** Ventral motor root from wild type mice showing many myelinated axons of different calibers. **D.** Ventral motor root from *dt^27J^* mice showing a mixture of myelinated and amyelinated axons of different calibers. Several large and intermediate caliber amyelinated axons are detected. The *dt* ventral motor root is smaller than the wild type counterpart and the axons are more compacted. Scale bar, 5 µm (all panels).

Previous studies have reported that *dt^27J^* peripheral sensory nerves exhibit hypomyelination, reduction in axonal caliber, and amyelinated axons [38]. Here, we explored the integrity of L1 dorsal and ventral spinal roots via toludine blue staining. In contrast to wild type controls (Fig. 5A), *dt^27J^* spinal roots had fewer sensory axons, and contained axonal swellings (Fig. 5B). Wild type ventral root analysis showed myelinated axons of different calibers (Fig. 5C), while *dt^27J^* ventral roots manifested amyelinated intermediate and large caliber axons (Fig. 5D). While normal axonal myelination of different axon calibers was present in *dt^27J^* ventral roots, amyelinated axons were evident in different regions of the ventral root. Upon quantification, the total number of motor axons within *dt^27J^* ventral roots was significantly lower than that in wild type ventral roots. A total of 1531 axons ±100.02 (SE, n = 3) within wild type ventral root cross-sections were recorded compared to 670 axons ±101.51 (SE, n = 3) within *dt^27J^* ventral root cross-sections (p = 0.02, Fig. 2D). Of the motor axon number reduction in *dt^27J^* ventral roots, large caliber axons (≥5 µm) were predominantly affected. Wild type ventral roots comprised 12.51% ±2.19 (SE, n = 3) large caliber axons, while *dt^27J^* ventral roots had fewer large caliber axons (7.89%±1.68; SE, n = 3, p = 0.03, Fig. 2E).

**Figure 6:**
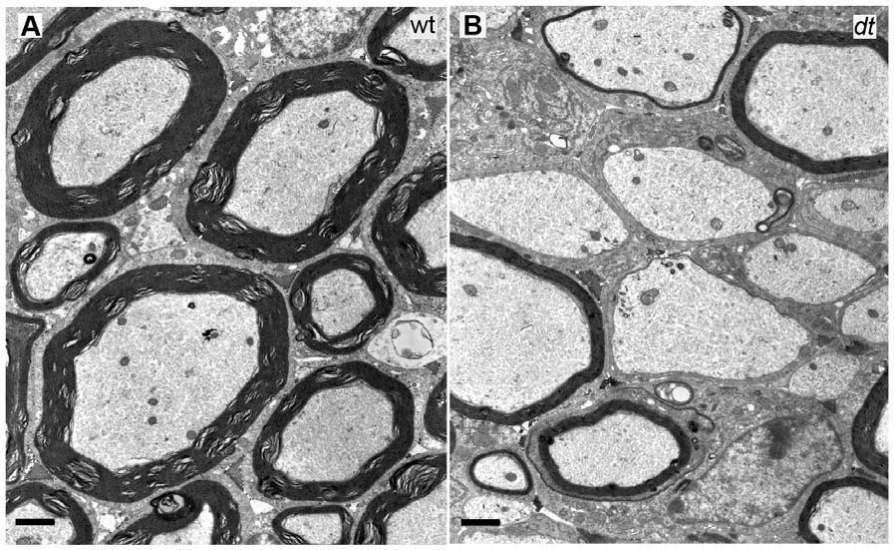
Ultrastructural analysis of ventral motor roots of the L1 spinal nerve of wild type and *dt^27J^* mice. Transverse sections of ventral motor roots from P15 wild type and *dt^27J^* mice were prepared for electron microscopy. **A.** Ventral motor root from wild type mice showing normal myelinated axons of different calibers. **B.** Ventral motor root from *dt^27J^* mice showing hypomyelinated and amyelinated axons of large and intermediate caliber. Scale bar, 1 µm.

To obtain a better understanding of the myelination defects, P15 wild type and *dt^27J^* spinal ventral roots were examined via electron microscopy. Other than a few amyelinated small caliber axons, we found that axons of different calibers – large, intermediate and small – manifested normal myelination in the wild type samples (Fig. 6A). In contrast, *dt^27J^* ventral roots of the L1 spinal nerve displayed numerous amyelinated axons of different calibers (Fig. 6B). Normal myelinated axons of different calibers were also present in the *dt^27J^* preparation.

### NMJ endplates from *dt^27J^* muscle are reduced in number and appear less mature

Initial observations of *dt* mice reported partial motor denervation of muscle and atrophy of some groups of muscle fibers [6]. To determine whether defects located more distally at the NMJ were present, we examined endplates of EDL and TA muscles in P15 mice. We first investigated whether a difference existed between the total number of wild type and *dt^27J^* TA and EDL endplates. We found a modest reduction (14.6%, 549±27 endplates, n = 3, p<0.05) in the total number of *dt^27J^* EDL endplates compared to wild type (643±24 endplates, n = 3). Consistent with this, the reduction observed in *dt^27J^* TA was 7.7% (2875±50 endplates, n = 3, p<0.01) in comparison to wild type TA muscle (3116±36 NMJs, n = 3). No difference in the number of endplates was observed in the *dt^27J^* diaphragm in comparison with wild type diaphragm (data not shown). While each EDL and TA *dt^27J^* myofiber had an endplate, there were fewer *dt* myofibers when compared to wild type myofibers (data not shown), and this could account for the reduction in endplate number.

**Figure 7:**
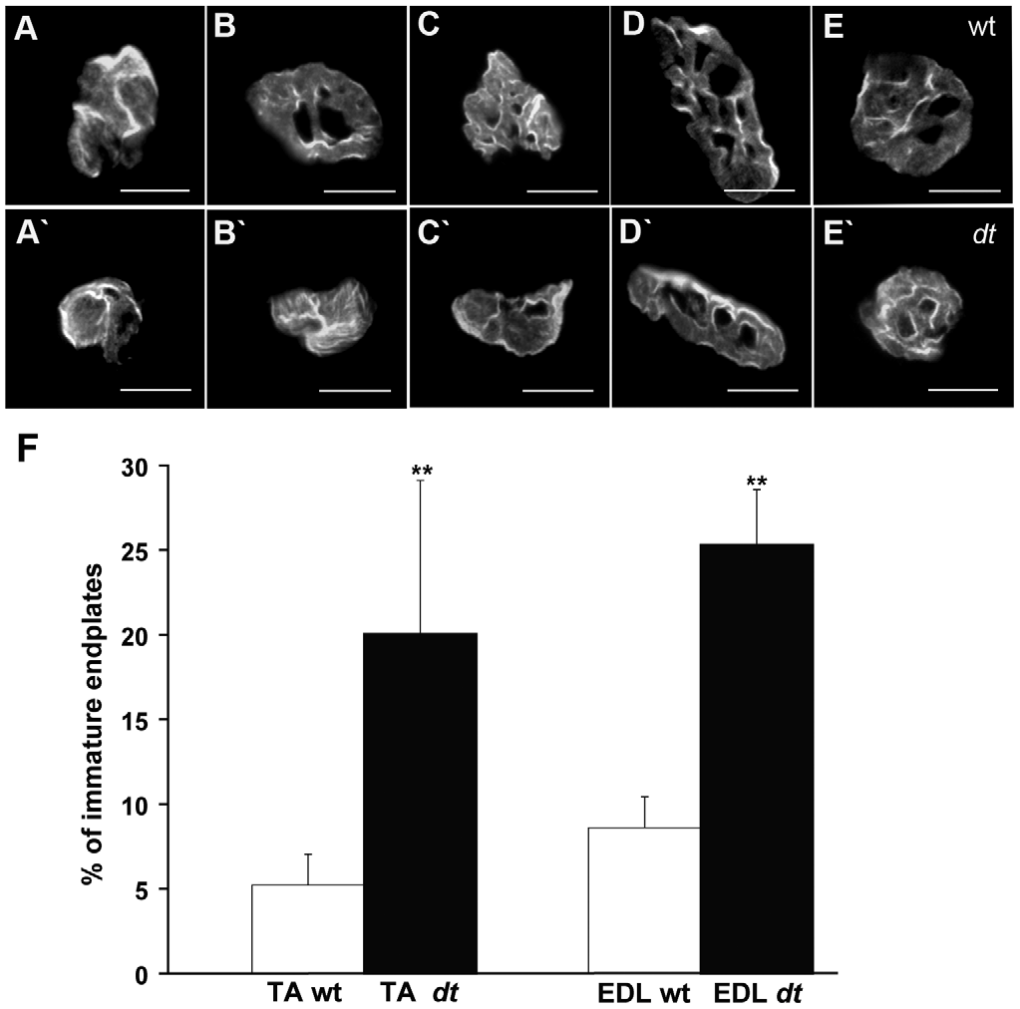
Endplates from *dt^27J^* mice are poorly developed. Representative photomicrographs showing NMJ endplate morphology in TA muscle at P15 from wild type **(A**–**E)** and *dt^27J^* **(A'**–**E')** mice. *dt^27J^* TA muscles show more immature NMJs (A'–B') when compared to wild type (A–E). The endplate morphology included two different patterns frequently observed – an elongated form with domains (D and D') and oval-like form with domains (E and E'). Scale bar, 15 µm. **F.** Graphical representation showing the percentage of immature endplates in TA and EDL muscles from wild type (n = 5) and *dt^27J^* (n = 5) mice at P15 (**p<0.01, student t-test).

The formation of mature NMJ endplates is established at approximately E17 and undergoes further structural and functional change during postnatal development. Maturity is achieved when endplates acquire the characteristic “pretzel-like” pattern with numerous folds (perforations) [41]. Morphological analysis (Fig. 7) of NMJ endplates revealed that 5.22% of endplates were immature (without perforations) in wild type TA muscle and 94.88% began to have one or more perforations. We observed 90% of NMJ endplates manifested two patterns – an elongated pattern and an oval-like pattern (Fig. 7D, E respectively), while the remaining 10% showed various patterns. This patterning was consistent between wild type and *dt^27J^* in TA muscle (Fig. 7D', E'). In contrast however, there was an increase in the percentage of immature endplates in both the TA and EDL muscles from *dt^27J^* mice (Fig. 7F). These results indicate an increase in the number of immature endplates (lacking perforations) in *dt^27J^* mice.

### Abnormalities at the NMJs in P15 *dt^27J^* mice include reduced axonal branching, pre-synaptic swellings, and motor neuron denervation

**Figure 8:**
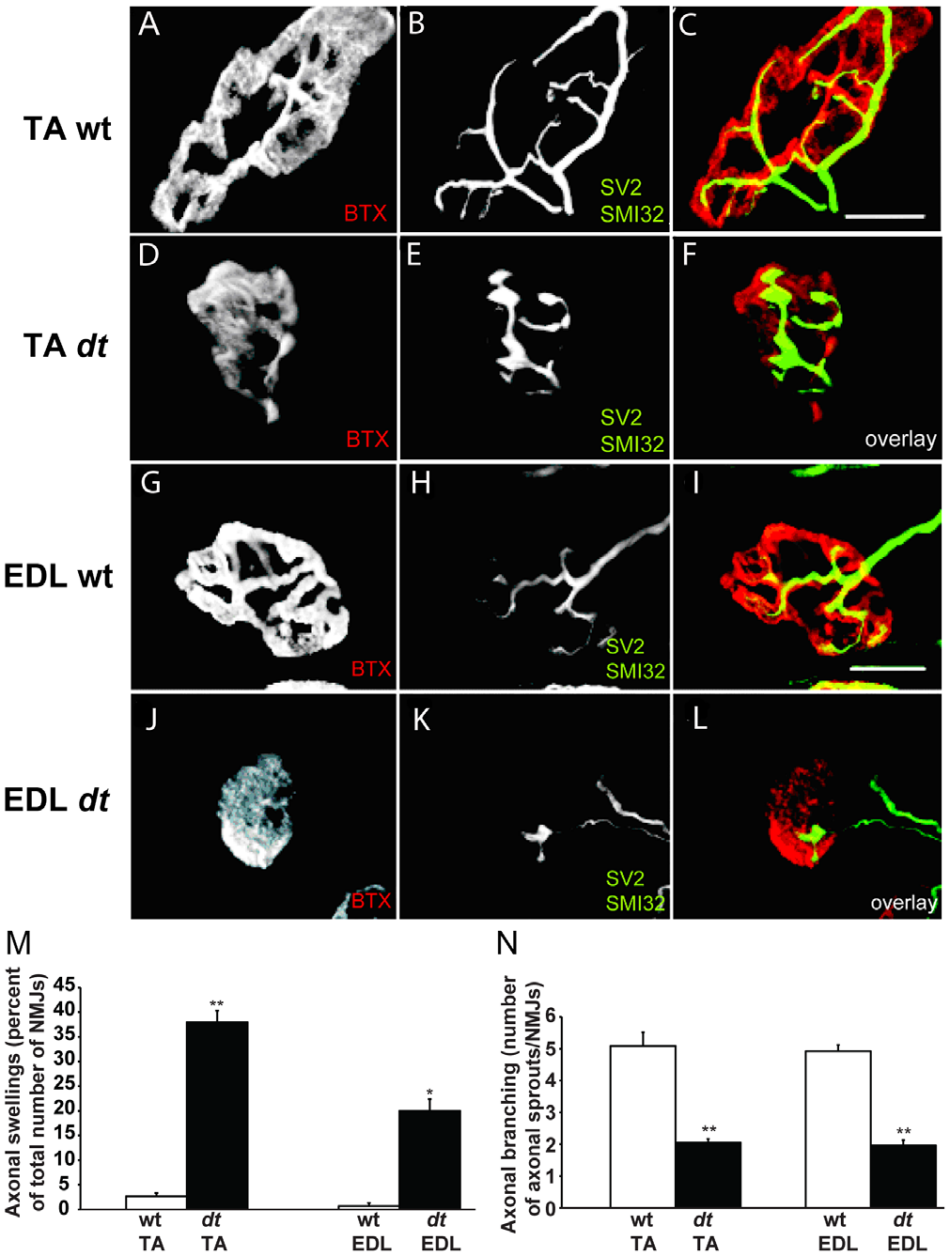
Pre-synaptic axonal swellings and reduced axonal sprouting at TA and EDL NMJs from *dt^27J^* pre-synaptic terminals. **A**–**L.** Representative confocal images of wild type (A–C) and *dt^27J^* (D–F) TA NMJs and wild type (G–I) and *dt^27J^* (J–L) EDL NMJs. NMJs are labeled with alpha-bungarotoxin (BTX, red in the merged image), the axons with SMI-312 (green) and the synaptic vesicles with SV2 (green). Scale bar, 50 µm. *dt^27J^* pre-synaptic terminals display fewer arbors and increased bulb-like swellings (F and L) whereas wild type pre-synaptic terminals exhibit extensive axon branching with no bulb-like swellings (C and I). *dt^27J^* endplates (D and J) appear less mature (fewer perforations) in comparison to wild type littermate endplates (A and G). **M.** The percentage of pre-synaptic swellings at TA/EDL NMJs in wild type (n = 3) and *dt^27J^* (n = 3) mice (TA NMJ, **p<0.01; EDL NMJ, *p<0.05, student t-test). **N.** Quantification of axon sprouting between wild type (n = 3) and *dt^27J^* (n = 3) at TA and EDL NMJs indicates reduced axon sprouting in *dt^27J^* NMJs (TA/EDL NMJ, **p<0.01, student t-test).

Based on the defects found at *dt^27J^* TA endplates in P15 mice, we decided to examine aspects of P15 TA and EDL NMJs, in particular motor neuron innervation, axonal branching and distal motor neuron integrity. Antibodies against NF protein and synaptic vesicles (SV2) were employed to visualize distal axons and nerve terminals, respectively. Acetylcholine receptors (AChRs) were stained with labeled bungarotoxin. Pre-terminal axons and nerve terminals of TA and EDL muscle from *dt^27J^* mice, but not from control littermates were characterized by axonal swellings (Fig. 8F and L). Approximately 40% of TA NMJs and 20% of EDL NMJs from P15 *dt^27J^* mice displayed bulb-like accumulation at nerve terminals (Fig. 8M). Motor endplates apposed to these terminals were small and structurally poorly developed (*i.e.*, less perforated) (Fig. 8D and J). Additionally, P15 TA and EDL NMJs from P15 *dt^27J^* mice exhibited reduced axonal branching, which accompanied the axonal swellings and the poorly developed endplates (Fig. 8A–L, and N). We did not detect poly-innervated endplates as previously described [6]. Collectively, these results suggest that axonal swellings within the distal nerve and poor terminal arborization are previously unappreciated aspects of *dt* pathology.

### Atrophy Muscle in *dt^27J^* mice

**Figure 9:**
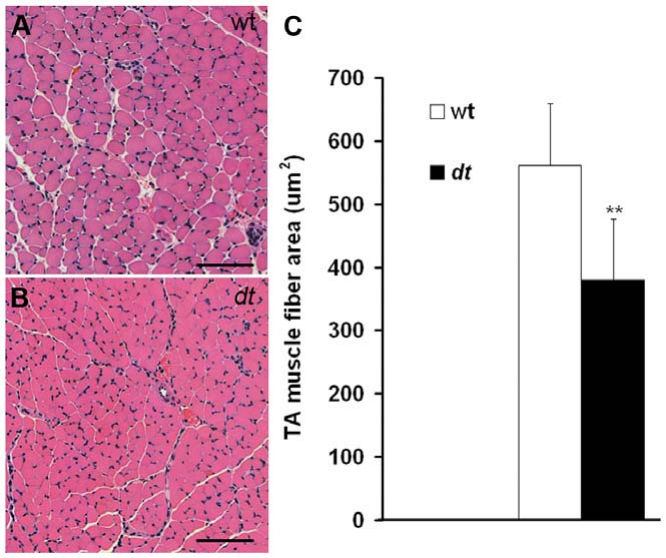
TA myofiber atrophy in *dt^27J^* mice at P15. TA myofibers in cross-section were stained with hematoxylin and eosin. **A.** Healthy myofibers from TA wild type muscle were observed under light microscopy. **B.** Myofiber atrophy in TA *dt^27J^* muscle is observed. Myofibers are smaller and many more nuclei are visible. **C.** A graphical representation showing the TA muscle fiber area in µm^2^ from wild type (n = 6) and *dt^27J^* (n = 6) mice revealed muscle atrophy in *dt^27J^* mice (** p<0.01, student t-test). Scale bars (A, B), 100 µm.

Seeing that P15 *dt^27J^* TA and EDL NMJs were morphologically abnormal, we sought to investigate whether these abnormalities would impact the integrity of the muscle. Using hematoxylin and eosin stain we found *dt^27J^* TA myofibers to be smaller (Fig. 9A, B). Muscle fiber size quantification revealed muscular atrophy in *dt^27J^* mice (Fig. 9C). The myofiber size was significantly reduced in *dt^27J^* TA muscle with a mean of 560.79 µm^2^±97.75 (SD, n = 6) compared to a 380.74 µm^2^±96.15 (SD, n = 6) in wild type TA muscle (**p<0.01). To assess fiber type grouping, immunostaining of mouse TA muscle sections with myosin heavy chain isoforms (type1, 2A, 2B and 2X) was performed. The analysis revealed no fiber type grouping in *dt^27J^* TA muscles (Fig. S1).

### Loss of alpha motor neurons in the *dt^27J^* brainstem

**Figure 10:**
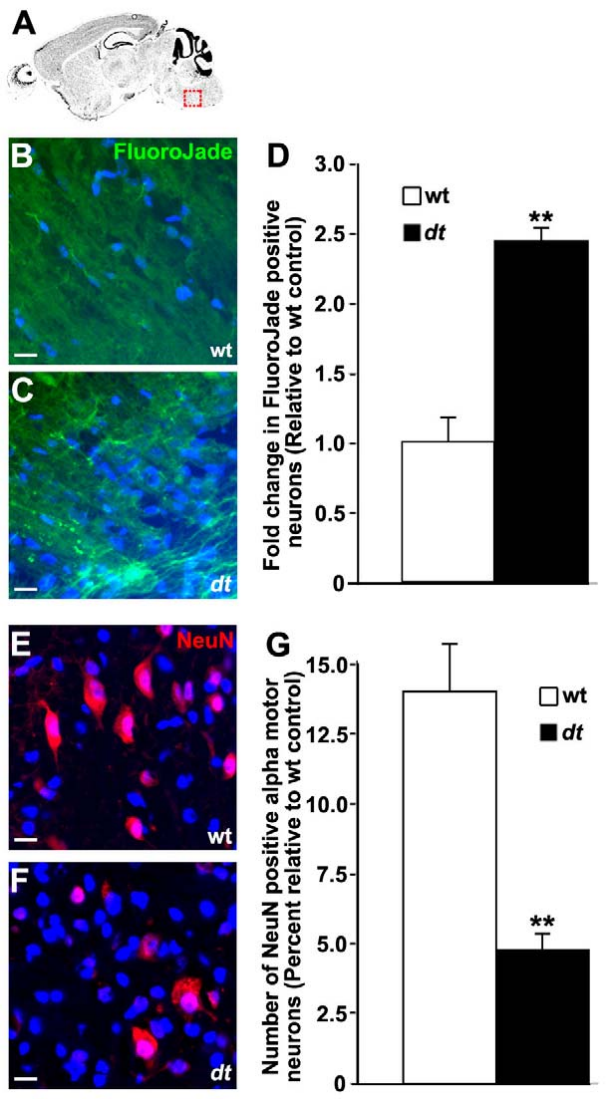
Motor neuron degeneration in the brainstem of *dt^27J^* mice. Sagittal sections from mouse brainstem (trigeminal nerve of the pons) identified in (**A**) of P15 wild type (**B**) and *dt^27J^* (**C**) mice were stained for degenerating neurons with Fluorojade B. **D.** Fold change in neurodegeneration in *dt^27J^* brainstem relative to wild type littermates is depicted (** p<0.01, student t-test). Sagittal sections from mouse brainstem of P15 wild type (**E**) and *dt^27J^* (**F**) mice were antigenically labeled for mature neurons with NeuN. Alpha motor neurons are identifiable by their larger size (greater than 9 µm, arrow) relative to other neuronal cell types (arrowhead). Nuclei were counter stained with DAPI to facilitate quantification. **G.** Quantification of percent alpha motor neurons yielded a decrease in neuron number in the trigeminal nerve of *dt^27J^* mice relative to wild type littermates (**p<0.01, student t-test). Scale bars, 10 µm.

In addition to the spinal cord, alpha motor neurons reside throughout the brainstem where they coordinate motor function via innervations of extrafusal muscle fibers. We thus evaluated the impact of dystonin function on alpha motor neuron survival in the trigeminal nerve of the pons, a region known to express high levels of dystonin [16]. FluoroJade staining, that specifically labels degenerating neurons [42], showed pronounced degeneration of neurons in the brainstem of *dt^27J^* mice (Fig. 10A–D). Quantification of FluoroJade positive neurons (Fig. 10D) yielded a 2.4 fold increase in degeneration in *dt^27J^* relative to wild type. To further evaluate whether this degeneration results in a loss of motor neurons, we quantified NeuN staining neurons in the brainstem of wild type and *dt^27J^* mice (Fig. 10E–G). Based on morphological analysis of NeuN positive cells, we found a decrease in alpha motor neuron cell number from 13% per 100 µm^2^ in wild type to 4% per 100 µm^2^ in *dt^27J^* (Fig. 10G) with correction for profile and section thickness according to the method of [36].

### Behavioral analysis reveals defective motor function in *dt^27J^* mice

Signs of weakness were observed in the forelimb and in the hind limbs of all *dt^27J^* mice and locomotor dysfunction was obvious at P15. Our motor examination revealed that dystonic pups could be affected to different degrees even within the same litter at P15. Severely affected *dt^27J^* mice (approximately 70% of all *dt^27J^* mice) were not able to grasp a flexible rod by their forelimbs at P15, but less affected *dt^27J^* mice (about 30% of all *dt^27J^* mice) were able to grasp the rod with their hind limbs for a few seconds. Nevertheless, compared to wild type littermates, the mutant mice had a decreased latency to fall (Table 3).

**Table 3:** Results from behavioral tests performed on wt and *dt^27J^* mice at P15.

Rod test				Righting reflex				Negative geotaxis			
wt		*dt*		wt		*dt*		wt		*dt*	
#	Meanlatencyto fall(sec±sd)	#	Meanlatencyto fall(sec±sd)	#	Rightingresponsetime	#	Rightingresponsetime(sec±sd)	#	+or–	#	+or–
1	15.0±3.8	6	3.4±0.6	11	0	16	6.8±1.3	21	–	26	+
2	19.0±5.2	7	2.6±0.9	12	0	17	11.2±3.5	22	–	27	+
3	10.2±2.7	8	3.0±0.7	13	0	18	4.0±1.4	23	–	28	+
4	22.4±5.3	9	4.2±0.8	14	0	19	8.4±0.5	24	–	29	+
5	16.6±3.9	10	4.8±0.8	15	0	20	4.4±1.3	25	–	30	+

#  =  mouse identification, values are expressed as mean ± standard deviation, 0 =  righting response occurs immediately, +  =  positive geotaxis, and –  =  negative geotaxis.

We also tested *dt^27J^* mice in other motor behavior tests. In the righting reflex test, mutant mice had a greater latency to right themselves as compared to wild type littermates when placed in a supine position (Table 3). Similarly, *dt^27J^* mice did not exhibit negative geotaxis and fell off an inclined grid, whereas wild type littermates were always able to turn themselves around and walk up the grid (Table 3). Collectively our behavioral analysis identified defective motor function in *dt^27J^* mice.

## Discussion

In the present study, we investigated motor neuron pathology in the spontaneous *dt^27J^* mouse mutant. We have assessed the integrity of L1 motor neurons and the NMJs of *dt^27J^* mice. Alpha motor neurons of the L1 spinal cord are reduced in number and show eccentric nuclei. Consistent with this, a significant reduction in number of motor axons, most notably large caliber motor axons, was noted. Accumulation of phosphorylated NFs within motor neuron perikarya and axons was also evident. Ultrastructural studies revealed that ventral root spinal axons of different calibers were hypo or amyelinated, implying a defect in Schwann cells. In addition, we show *dt^27J^* TA and EDL NMJs are poorly developed. Pre-synaptically, the defects are defined by denervated endplates, axonal swellings in the nerve terminals and poor terminal arborization, while post-synaptically poorly developed endplates were noted. The NMJ defects are coupled with TA muscle atrophy, but an absence of fiber type grouping. Finally, brain stem analysis revealed a 2.4 fold increase in degenerating neurons coupled with a decrease in motor neuron number. The observed neuromuscular defects were accompanied by a deficit in motor behavior. Although some of the defects observed may be attributed to the overall smaller size of *dt^27J^* mice compared to wild type littermates, it is nevertheless clear that neuromuscular defects likely contribute to the *dt^27J^* pathology and our study identifies a critical role for dystonin outside of sensory neurons. It is possible that there is a second mutation within the *dt^27J^* mice that is responsible for the observed motor neuron pathology. However, this is unlikely since we have yet to observe a segregation of the sensory and motor neuron defects in *dt^27J^* mice. The latter would have been expected if two different genetic loci were involved. Furthermore, motor neuron pathology has been observed in other *dt* mouse strains [6], [24].

### Structural abnormalities of the NMJ: a new *dt* pathology

The *dt* pathology has long been defined as a sensory neuropathy. While it has been shown that dystonin mRNA expression levels are equivalent in alpha motor neurons and sensory neurons of the adult spinal cord [16], and that *dt* mice resemble a neuromuscular disorder, it is surprising the NMJ has not been studied in *dt* mice. Here, we sought to fill this gap by studying *dt^27J^* NMJs. Our analysis indicates that NMJ defects are present in TA and EDL muscle at the phenotype stage (P15) in these mice. Abnormalities include poor terminal arborization, axonal swellings in nerve terminals and/or pre-terminal axons, and poorly developed, morphologically immature AChR clusters that never attain the complex pretzel-shaped structures seen in wild type animals. The formation of NMJs, which involves nerve-muscle contact, intramuscular nerve branching and neuronal survival, requires reciprocal signals from nerve and muscle [43]. Indeed, muscle-derived retrograde factors mediate motor neuron survival, remodeling and activity-induced changes at the NMJ [44]. Thus it was of interest to observe that muscle endplates are poorly developed in various *dt^27J^* muscles.

It should be noted that in addition to the pathological features at the endplates, previous work has shown cytoskeletal disorganization in *dt* skeletal muscle [45]. How or if immature endplates contribute to the motor neuron defects in *dt* mice remains to be determined. Poorly developed endplates described in the present study likely occur via aberrant cytoskeletal organization, hence leading to insufficient crosstalk between *dt^27J^* muscle endplates and motor nerve terminals. This is important as crosstalk, mediated by muscle-agrin signaling, initiates axon branching [46].

The motor pathology reported here including denervation and decreased axonal branching could also be brought on via intrinsic mechanisms. Previous studies have noted that motor neurons effectively innervate *dt* muscle. Only later in life at approximately one month of age has denervation been reported in *dt* mice [6]. It is safe then to presume that axon path-finding is not perturbed in *dt* motor neurons. Rather, loss of dystonin protein, which results in disorganized cytoskeleton, leads to reduced pre-synaptic arbors and eventual dying-back of motor neurons. Mechanisms mediating reduced axonal sprouting and dying-back are unknown. However, *dt* motor neurons contain focal swellings consisting of disorganized NFs, microtubules and abundant secondary lysosomes, which may impede axonal trafficking [24]. In fact, sciatic nerves from *dt^27J^* mice have a fast bi-directional transport impairment of acetylcholinesterase [23]. Impeded transport by NF accumulation or aberrant cytoskeleton organization may be assisting this dying-back event. The NF accumulation within terminal axons likely contributes to the paucity of terminal arborization. Indeed, similar pathology has been shown in NMJs of spinal muscular atrophy mice. Notably, the loss of survival motor neuron gene leads to pre-synaptic NF accumulation and reduced axonal sprouting and eventual dying-back [47].

Whether the motor defects shown here are intrinsically caused by the loss of dystonin or, alternatively, are secondary to sensory neuron degeneration, remains unclear. Moreover, whether or not motor neurons are affected through a lack of crosstalk between muscle and motor neurons remains to be established. Interestingly, null mutations within the *kakapo* gene, the *Drosophila* ortholog of ACF7/MACF1 which shares high sequence homology with dystonin [48], results in NMJ abnormalities, notably reduced terminal branches [49]. Moreover, rapsyn – a post-synaptic scaffolding protein, which clusters AChRs at the endplate – associates with the actin binding domain of ACF7/MACF1 in skeletal muscle [50]. The one detractor of this study was that interactions between rapsyn and ACF7/MACF1 were observed in a non-muscle system. Nevertheless, the fact that a plakin protein family member, ACF7/MACF1, interacts with NMJ proteins and the loss of dystonin expression yields immature endplates leads us to speculate that dystonin may also have a role in the formation and/or maintenance of endplates.

### Schwann cell defects: a contributor to *dt^27J^* pathology

We show that ventral roots of *dt^27J^* mice exhibit hypo- and amyelinated axons. Previous studies have shown that dystonin is an essential component of the Schwann cell cytoskeleton and loss of dystonin expression causes hypo/amyelinated peripheral nerves [38]. It is tempting to associate the Schwann cell defects seen here with the NMJ abnormalities. For instance, Schwann-cell processes are known to cap the nerve terminals, thereby providing insulation and perhaps trophic sustenance. In addition, Schwann cells ensheathe motor axons and pre-terminals allowing proper saltatory conduction along the nerve fiber and signal transmission to the muscle [51]. Mechanisms mediating the defective myelination in *dt^27J^* mice are unknown. We hypothesize that because Schwann cells undergo extensive cytoskeletal rearrangements during the myelination process [52], and dystonin is a cytoskeletal linker protein, it is likely that loss of dystonin perturbs cytoskeletal-driven axon ensheathment. Alternatively, disruption in the composition of *dt* axonal proteins, due to axonal swellings for instance, may impede signaling between the axon and Schwann cell, thus leading to improper myelination. However, it seems unlikely that this latter possibility contributes to defective myelination as Schwann cell defects in *dt* mice are intrinsic [38]. Finally, it is important to note that myelination has a profound effect on axon caliber, distal nerve terminals and long-term survival [53], [54], [55]. The absence of ventral root myelination seen here may account for the reduced number of large caliber axons and distal defects.

The *dt* pathology has classically been described as a sensory neuropathy. Here, we have shown that in addition to the sensory neuron defects, the *dt^27J^* mouse displays motor neuron and NMJ pathologies. When considering the fact that *dt* mice die at weaning, the importance of dystonin in various tissues becomes grossly evident.

## Supporting Information

Figure S1 **Absence of fiber type grouping in TA muscle of *dt^27J^* mice**. Cryostats sections (12 µm) of TA muscles obtained from wild type (**A**,**C**, **E** and **G**) and *dt^27J^* (**B**, **D**, **F** and **H**) mice at P15 were immunostained with laminin (red) and myosin heavy chain (green) (type 1 (A,B), type 2A (C,D), type 2B (E,F) and type 2X (G,H). No fiber type grouping was observed. Scale bars (A–H), 20 µm.
(TIF)
