# Supplementary figures and images for: Motor Unit Abnormalities in Dystonia musculorum Mice
Source: PLoS One. 2011 Jun 15;6(6):e21093. doi: 10.1371/journal.pone.0021093 (PMC3115977; doi:10.1371/journal.pone.0021093)

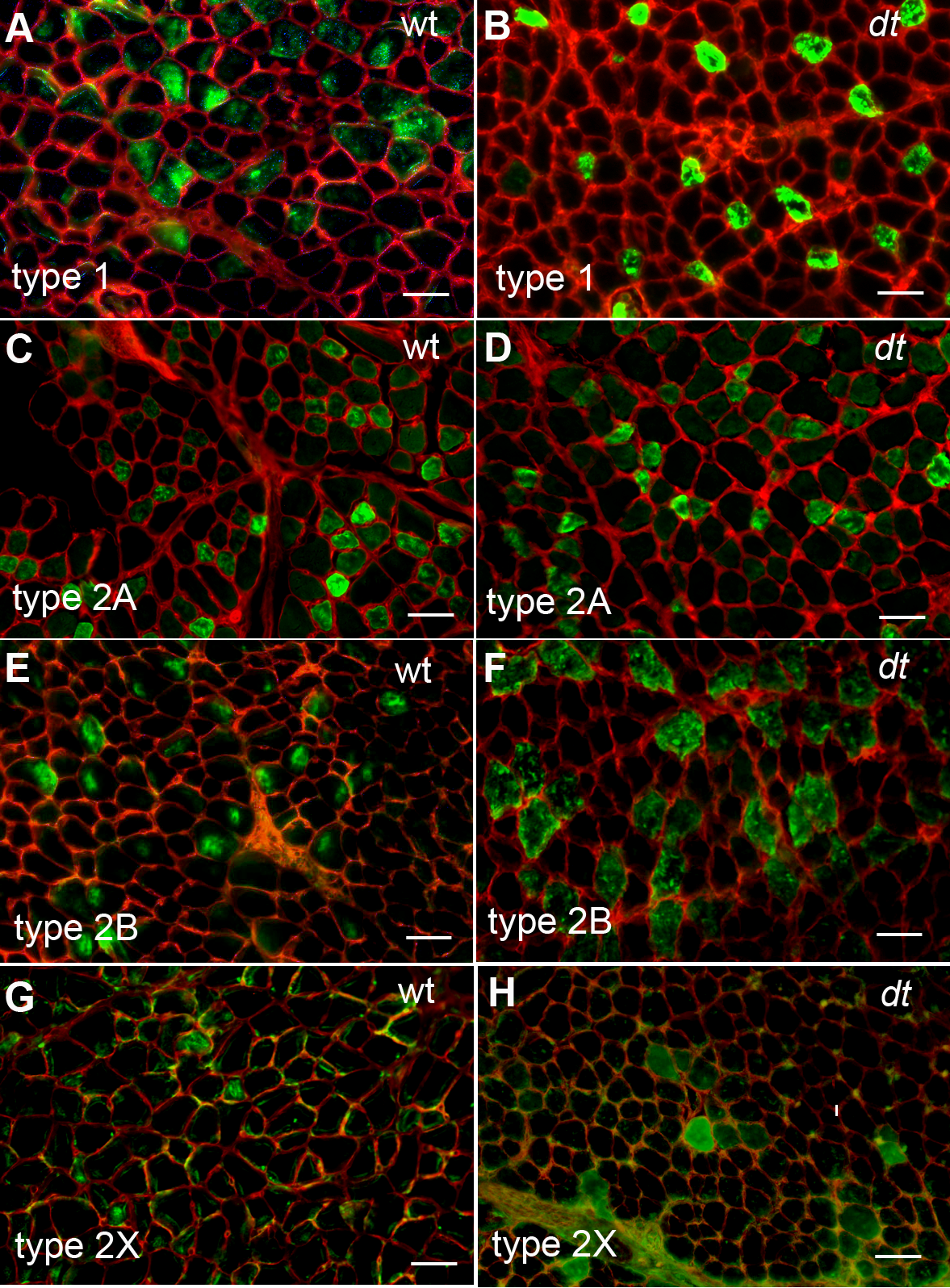

Supplement: Figure S1 — Absence of fiber type grouping in TA muscle of dt27J mice. Cryostats sections (12 µm) of TA muscles obtained from wild type (A,C, E and G) and dt27J (B, D, F and H) mice at P15 were immunostained with laminin (red) and myosin heavy chain (green) (type 1 (A,B), type 2A (C,D), type 2B (E,F) and type 2X (G,H). No fiber type grouping was observed. Scale bars (A–H), 20 µm. (TIF) [file pone.0021093.s001.tif]
